# Unique photosynthetic electron transport tuning and excitation distribution in heterokont algae

**DOI:** 10.1371/journal.pone.0209920

**Published:** 2019-01-09

**Authors:** Gunvor Bjerkelund Røkke, Thor Bernt Melø, Alice Mühlroth, Olav Vadstein, Atle M. Bones, Martin F. Hohmann-Marriott

**Affiliations:** 1 Department of Biotechnology, Norwegian University of Science and Technology, Trondheim, Norway; 2 Department of Physics, Norwegian University of Science and Technology, Trondheim, Norway; 3 Department of Biology, Norwegian University of Science and Technology, Trondheim, Norway; Southern Cross University, AUSTRALIA

## Abstract

Heterokont algae are significant contributors to marine primary productivity. These algae have a photosynthetic machinery that shares many common features with that of Viridiplantae (green algae and land plants). Here we demonstrate, however, that the photosynthetic machinery of heterokont algae responds to light fundamentally differently than that of Viridiplantae. While exposure to high light leads to electron accumulation within the photosynthetic electron transport chain in Viridiplantae, this is not the case in heterokont algae. We use this insight to manipulate the photosynthetic electron transport chain and demonstrate that heterokont algae can dynamically distribute excitation energy between the two types of photosystems. We suggest that the reported electron transport and excitation distribution features are adaptations to the marine light environment.

## Introduction

Heterokont algae have emerged as the result of a secondary endosymbiotic event [1,2] and dominate carbon fixation within the oceans [3,4]. However, the features that make heterokont algae so successful in the marine environment remain unresolved. Although separated by more than a billion years of evolution from a common ancestor, the photosynthetic machinery of Viridiplantae (green algae and land plants) and heterokont algae retains common features [2]. Both groups of organisms have two types of photosystems, which are linked by an electron transport chain that includes a pool of plastoquinone molecules. Another common feature is the presence of light-harvesting complexes, which house chlorophylls and carotenoids. There are, however, differences between Viridiplantae and heterokont algae in the type of chlorophylls and carotenoids they possess, as well as in the proteins involved in regulating light-harvesting and photoprotection [5,6]. A particularly interesting question is how these organisms utilize differences in their photosynthetic machineries to respond to the environmental conditions they encounter.

Viridiplantae dominate primary production on land, while heterokont algae dominate primary production in marine environments. Compared to *terra firma*, the light environment in the oceans is more dynamic, with wave-induced lensing exposing algae to rapid variations in light intensity [7,8]. It has been established that Viridiplantae can respond to high light by redistributing light-harvesting complexes from photosystem II (PSII) to photosystem I (PSI) [9]. This “state transition”, which in Viridiplantae is activated by a reduced plastoquinone pool, has not been demonstrated in heterokont algae, where a pH-dependent conversion of carotenoids [10] that is also present in Viridiplantae [11], is known to dissipate excess excitation energy [12,13]. In addition to forming a light-dependent pH-gradient, some green algae, including *C*. *reinhardtii* [14] and heterokont alga *P*. *tricornutum* [15,16] also maintain a pH-gradient during darkness, by oxygen-dependent chlororespiration.

Assessment of chlorophyll fluorescence, using a set of techniques originally developed for Viridiplantae, can provide exquisite insights into the photosynthetic electron transport chain and can be used to assess the photosynthetic performance [17]. It has been demonstrated that the maximum fluorescence yield can be obtained after applying a bright (saturating) light pulse that completely reduces the pool of electron acceptors (the plastoquinone pool), which is accepting electrons from PSII. As the plastoquinone pool is in equilibrium with the primary modulator of chlorophyll fluorescence, the PSII-bound Q_A_ [18], a complete reduction of the plastoquinone pool guarantees that Q_A_ molecules in all reaction centers are reduced, and thus that the maximum fluorescence yield is achieved [19]. Surprisingly, a detailed interpretation of chlorophyll fluorescence recently revealed that short, bright light pulses have a different effect on the electron transport chain in the heterokont alga *N*. *oceanica* than in Viridiplantae. While bright light pulses completely reduce the plastoquinone pool in Viridiplantae, bright light pulses applied to *Nannochloropsis* fail to do so [20]. This observation has potentially important implications for assessing photosynthetic performance that relies on achieving the maximum fluorescence yield as a reference point [21,22]. Furthermore, if the photosynthetic electron transport chain in heterokont algae also remains oxidized during longer exposures to high light, research into photoprotection mechanisms in this group of organisms has to be reinterpreted, and may uncover a novel light protection strategy. In this study, we set out to clarify the effect of high light exposure on the diatom *Phaeodactylum tricornutum* and the Eustigmatophyte *Nannochloropsis oceanica*, both marine heterokont algae.

## Results

**Fig 1:**
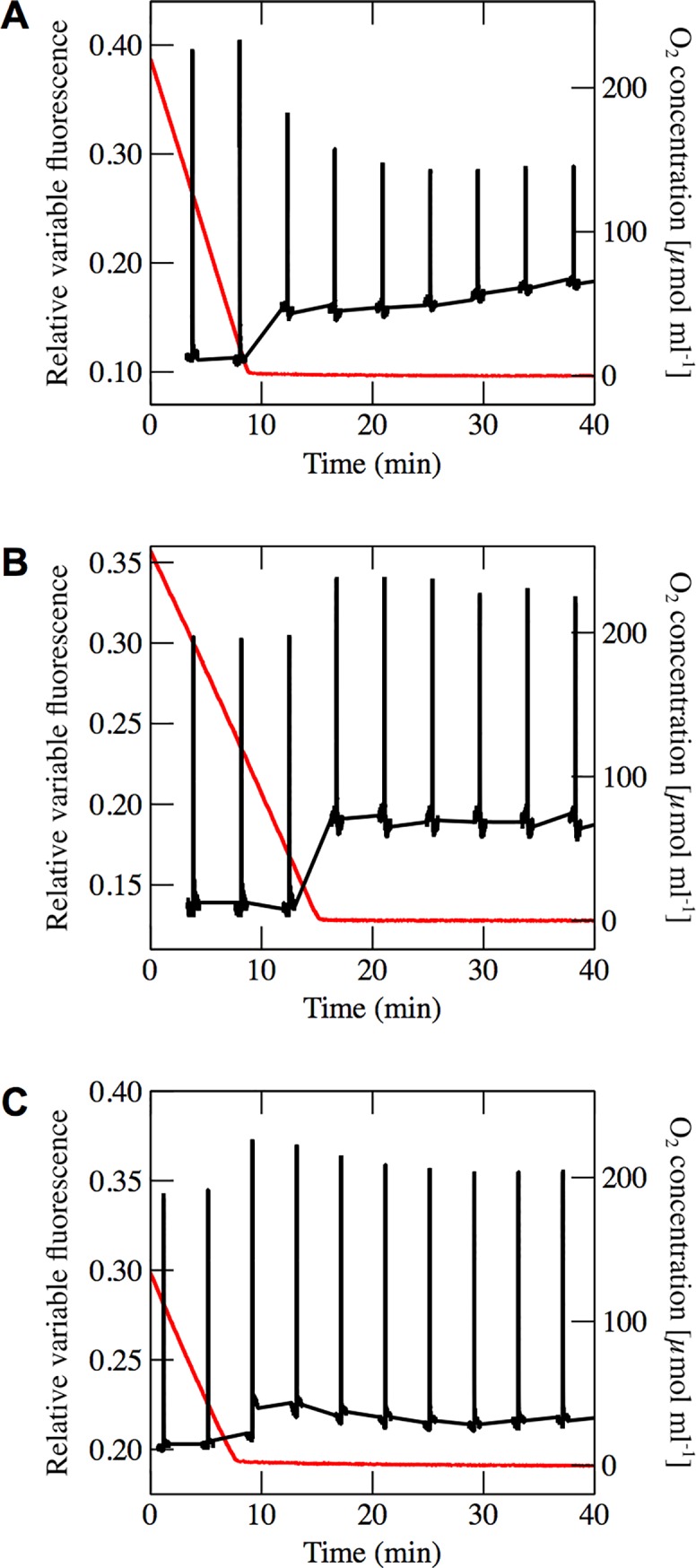
Variable fluorescence recorded during the shift from aerobic to anaerobic conditions. Variable fluorescence for (**A**) *C*. *reinhardtii*, (**B**) *N*. *oceanica* and (**C**) *P*. *tricornutum*. Black lines show variable fluorescence, and red lines show oxygen concentration, which was recorded simultaneously.

In order to determine whether bright light pulses completely reduce the plastoquinone pool, and thus induce the maximum fluorescence yield, we employed a reference condition that is known to result in a reduced photosynthetic electron transport chain. In darkness and in the absence of oxygen, metabolically generated electrons accumulate within algal cells. This leads to accumulation of electrons on electron carriers within the photosynthetic electron transport chain, including the plastoquinone pool [23,24]. If light pulse-induced fluorescence yield in the presence and absence of oxygen are identical, then the plastoquinone pool is reduced in both conditions. Using this assay, we observe that the light pulse-induced fluorescence yield in the Viridiplantae model organism *Chlamydomonas reinhardtii* is diminished in anaerobic conditions (Fig 1A) due to the state transition-associated move of light-harvesting complexes from PSII to PSI [25]. Conversely, the light pulse-induced fluorescence yield in *N*. *oceanica* and *P*. *tricornutum* increases in oxygen-free conditions (Fig 1A and 1B). We also investigated the effect of different light pulse intensities on the maximum fluorescence yield (S1 Fig) and Q_A_^-^ oxidation after light pulses in *P*. *tricornutum*. In these experiments, we used the cytochrome *b*_6_*f* inhibitor DBMIB as an alternative to anaerobic conditions to guarantee the reduction of the plastoquinone pool. DBMIB binds to the Q_o_ site of the cytochrome *b*_6_*f* complex, and thereby disables the Q-cycle and electron donation to plastocyanin. Light pulses applied in the presence of DBMIB therefore leads to complete reduction of the PQ pool and maximum fluorescence yield. This effect has been established for *C*. *reinhardtii* [26–28], *N*. *oceanica* [29–31] and *P*. *tricornutum* [32,33]. While DBMIB has been shown to have a quenching effect on chlorophyll fluorescence [34], this effect is constant, as samples were pre-incubated with DBMIB and the concentration did not change during the experiment.

Comparing untreated cells to DBMIB-treated cells indicates that in untreated cells in aerobic conditions the maximum fluorescence yield is not reached, not even at very high light intensities (S1 Fig). That the plastoquinone pool is not completely reduced can also be seen when investigating decay rates following a light pulse (S1 Fig). No matter by how much the light intensity of the light pulse is increased, the decay rates in DBMIB-treated cells was always lower, and the maximum fluorescence always higher than in untreated cells, except for in *C*. *reinhardtii*, where the light pulses were shown to yield a more or less reduced plastoquinone pool at higher light intensities (see S1 Discussion of S1 Fig). This conclusion is also in line with results obtained by analysing the fluorescence kinetics after light pulses. When the plastoquinone pool was reduced in the presence of DBMIB, the fluorescence decay rates were similar to the decay rates obtained in anaerobic conditions, even at low light intensities.

Similarly the decay rates of untreated cells at low chlorophyll concentration, even at very high light intensities, were similar to those obtained in aerobic conditions when a higher chlorophyll concentration was used (S1 Fig). Similar experiments to the one here presented for *P*. *tricornutum*, have also been published for *N*. *oceanica* and *C*. *reinhardtii* [20].

We can therefore conclude that routinely applied bright light pulses are not able to completely reduce the plastoquinone pool of the heterokont algae *N*. *oceanica* and *P*. *tricornutum* in the presence of oxygen (Fig 1B and 1C).

The obvious question arising from our initial observation of the effect of short light pulses is what effect a longer light exposure has on the reduction state of the plastoquinone pool, as it is–in analogy to Viridiplantae–generally assumed that the plastoquinone pool is reduced under high light intensities. Furthermore, we will determine whether modulating the reduction state of the photosynthetic electron transport chain induces a redistribution of excitation energy between the two photosystems in heterokont algae. This question has been previously addressed in the heterokont algae *P*. *tricornutum* [35], concluding that observed changes in fluorescence yield are not caused by a changing association of light-harvesting complexes between the two photosystems.

**Fig 2:**
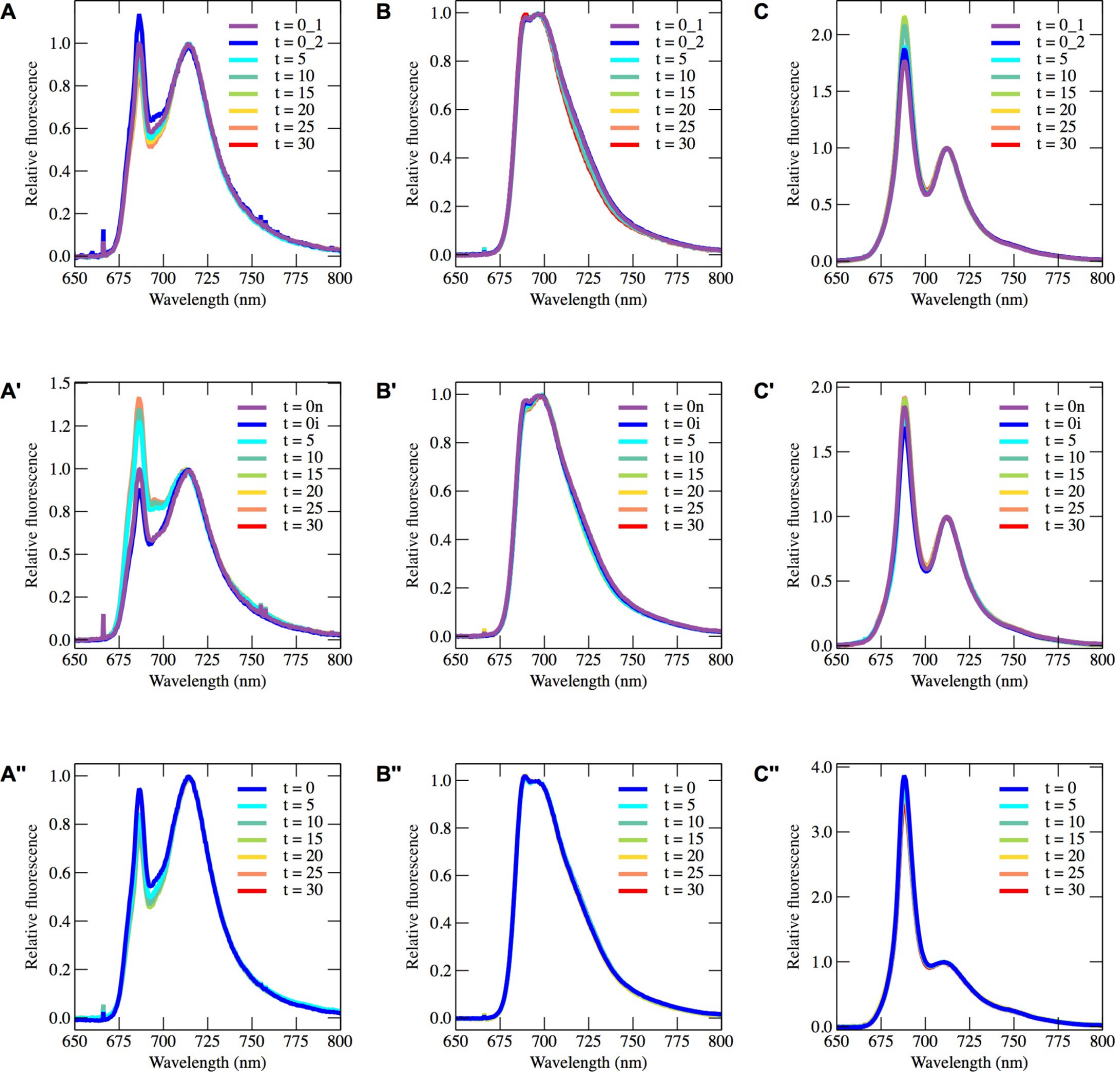
Raw spectra recorded by 77 K spectroscopy. 77 K fluorescence spectra from *C*. *reinhardtii* (**A**), *N*. *oceanica* (**B**) and *P*. *tricornutum* (**C**) recorded after exposure to high light (**A**, **B**, **C**), exposure to high light in the presence of DCMU (**A'**, **B'**, **C'**) and during oxygen-depletion in the dark (**A''**, **B''**, **C''**) using a 435 nm LED for excitation. All spectra have been baseline-corrected and normalized to the peak wavelength of PSI in each species.

**Fig 3:**
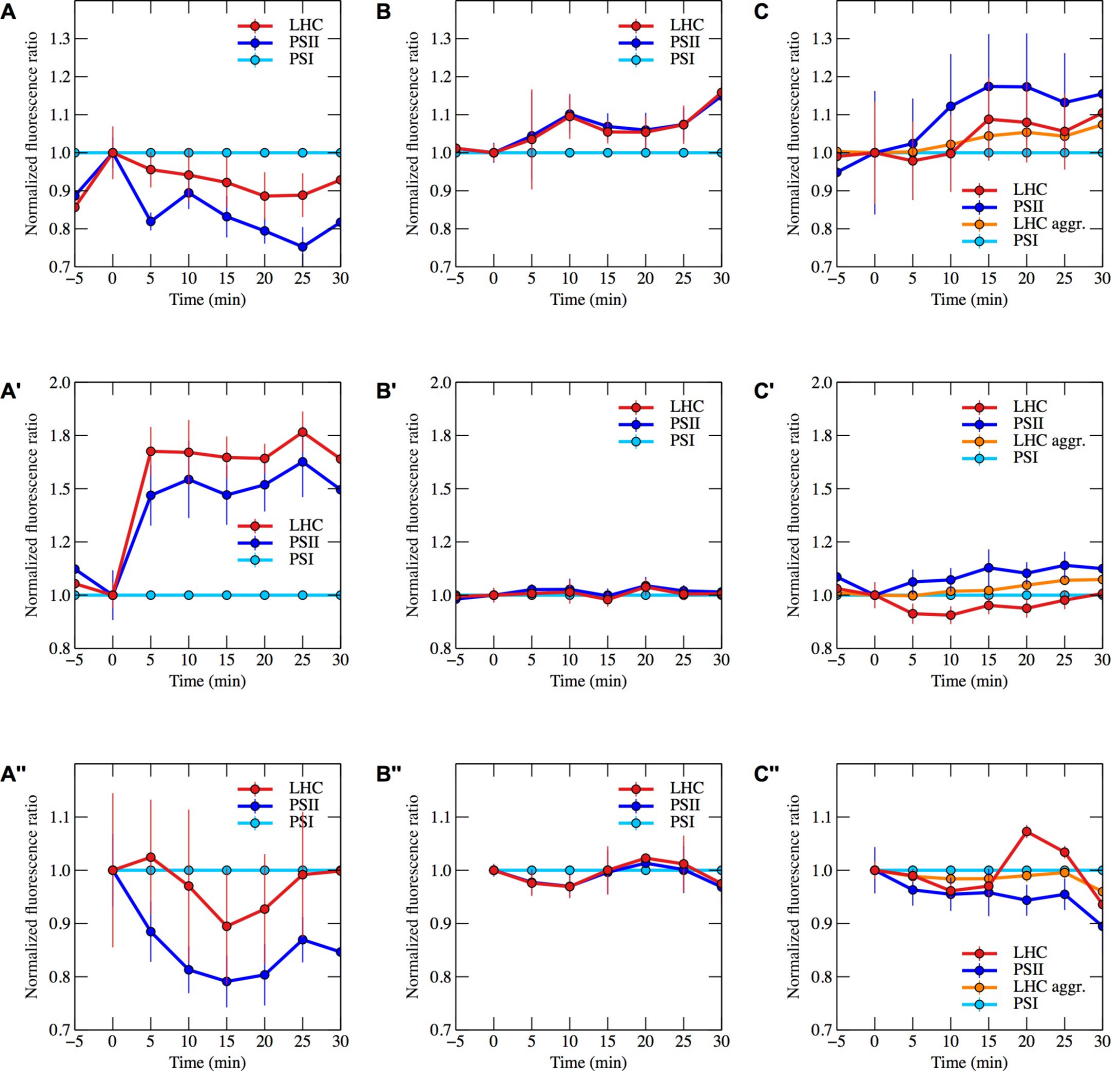
Fluorescence ratios for light harvesting complexes and PSII over time. Fluorescence ratios normalized to PSI over time during high light (**A**, **B**, **C**), high light in the presence of DCMU (**A’**, **B’**, **C’**) and during dark, anaerobic conditions (**A”**, **B”**, **C”**). (**A**, **A’**, **A”**) show ratios for *C*. *reinhardtii*, (**B**, **B’**, **B”**) show ratios for *N*. *oceanica* and (**C**, **C’**, **C”**) show ratios for *P*. *tricornutum*. Fluorescence spectra for the fluorescent components located in each organism, which led to the selection of the wavelengths representing PSII, PSI and light harvesting complexes, are shown in Fig 4.

**Fig 4:**
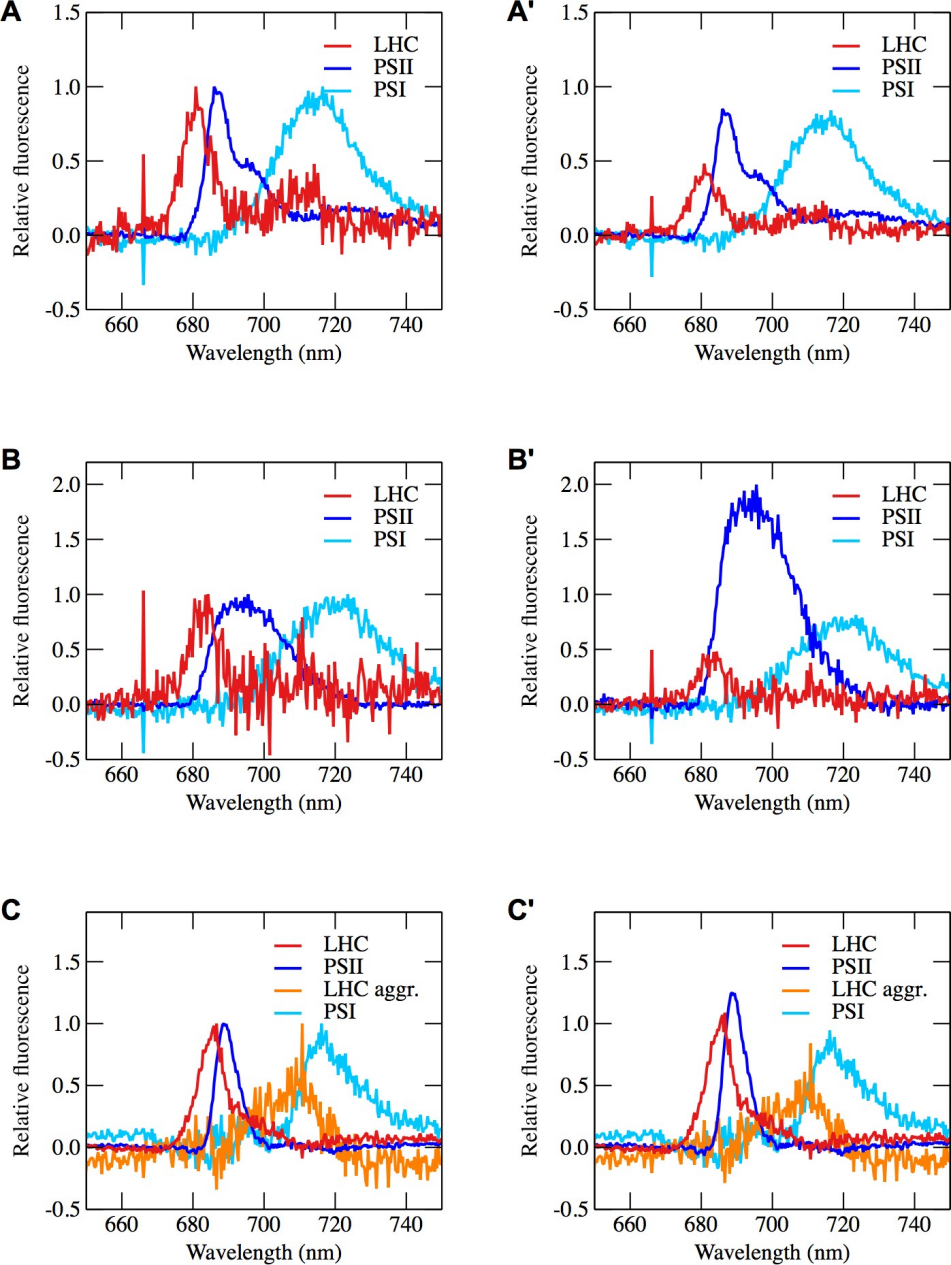
Fluorescent components found to be present in *Chlamydomonas*, *N*. *oceanica* and *P*. *tricornutum*. Calculated fluorescence spectra for photosynthetic components comprising fluorescence emission spectra at 77 K of (**A**) *C*. *reinhardtii*, (**B**) *N*. *oceanica*, and (**C**) *P*. *tricornutum*. In panels (**A**, **B**, **C**), the fluorescence spectra have been normalized to unity, while in panels (**A’**, **B’**, **C’**), the size of the respective fluorescing components correspond to their contribution to the overall fluorescence spectrum. The amplitude of the fluorescing components change throughout the experiments, but to give an example of their contribution in a spectrum, the reference sample of the high light experiments performed on *C*. *reinhardtii*, *N*. *oceanica* and *P*. *tricornutum* were chosen. The fluorescence components were calculated as shown in **S2 Fig**.

The current view in the model organisms *P*. *tricornutum* and other heterokont algae, is that chlorophyll fluorescence yield is modulated by pH-dependent mechanisms [12]. As chlorophyll fluorescence at room temperature cannot distinguish pH-dependent excitation quenching from other possible modes of quenching, such as a state transition, we chose to obtain spectral information of the fluorescence emission at 77 K (raw spectra shown in Fig 2, fluorescence ratios shown in Fig 3). This technique does not have as long a tradition of use in heterokont algae as it does in Viridiplantae [36], and we were initially taken aback by the dynamic behaviour we report and interpret here for the first time. These spectral dynamics allowed us to assign spectral components (Fig 4, see S2 Fig for a detailed demonstration of the procedure) that will be used to further discuss excitation distribution dynamics (Fig 3).

### Assigning identities to fluorescent components

The wavelengths used to indicate different fluorescent components were determined by deconvoluting 77 K fluorescence spectra (an example of such a deconvolution is shown in S2 Fig).

The fluorescence maxima of the fluorescent components found in *C*. *reinhardtii* were located at 680 nm, 686 nm and 716 nm (Fig 4A and 4A’) and were assigned to light harvesting complexes (LHCs) [37], PSII [38] and PSI [39], respectively. For *N*. *oceanica*, we identified fluorescent components at 684 nm, 695 nm and at 723 nm (Fig 4B and 4B’), and assigned these components to LHCs [40], PSII [40] and PSI [40,41].

For *P*. *tricornutum*, four components were identified with peak wavelengths at 686 nm, 688 nm, 710 nm and 715 nm (Fig 4C and 4C’). The component with a peak wavelength at 688 nm was assigned to PSII [42], and the 715 nm component was assigned to PSI due to spectral similarities with other diatom PSI, for example *Chaetoceros gracilis* [43] and *Cyclotella meneghiniana* [44]. The spectral component peaking at 710 nm is likely due to fluorescing light harvesting complex-aggregates, which have been demonstrated in diatoms, and particularly in *P*. *tricornutum* [5,45–47]. Isolated fucoxanthin chlorophyll *a*/*c* binding proteins (FCPs) from *P*. *tricornutum* have their 77 K fluorescence maximum at 680 nm [40], while the 77K fluorescence maximum for FCPs from *Chaetoceros gracilis* has been found at 683 nm [48]. The fluorescent component having a fluorescence maximum at 686 nm does not match fluorescence components that have been previously reported for isolated diatoms. This fluorescence emission may be due to highly fluorescent red-shifted emissions by fucoxanthin-chlorophyll binding proteins (FCPs) as recently described in the diatom *Cyclotella meneghiniana* [49].

### Modulation of the plastoquinone pool reduction state by high light

Exposure of *N*. *oceanica* and *P*. *tricornutum* to high light intensities results in a redistribution of excitation energy between the two types of photosystems. Upon illuminating these heterokont algae, the excitation is directed towards PSII (Fig 3B and 3C). This favouring of PSII indicates that the photosynthetic electron transport chain requires electron input, suggesting that the plastoquinone pool is largely oxidized. The response to high light is therefore opposite to what is observed in Viridiplantae, such as *C*. *reinhardtii*, where the plastoquinone pool is reduced during the initial exposure to bright light and excitation consequently is directed away from PSII (Fig 3A).

### Oxidation of the plastoquinone pool by DCMU treatment under high light

Exposure to the herbicide DCMU blocks electron generation by PSII, and in combination with light, leads to oxidation of the plastoquinone pool. The DCMU-treated and light-exposed heterokont algae, as well as *C*. *reinhardtii*, show a physiological response consistent with an empty plastoquinone pool by transferring excitation to the electron-generating PSII (Fig 3A’, 3B’ and 3C’). The magnitude of change in the fluorescence signal in the heterokont algae is, however, small compared to in *C*. *reinhardtii*. This is consistent with the assumption that the plastoquinone pool in the heterokont algae is mostly oxidized in dark aerobic conditions, while being partly reduced in *C*. *reinhardtii*. The plastoquinone pool of *C*. *reinhardtii* is also more reduced if acetate is included in the growth and experimental medium, as was the case in our experiments.

### Reduction of the plastoquinone pool by oxygen depletion

Exposure of algal cells to oxygen-free conditions in the dark results in an *in vivo* redox titration, as electrons accumulate within the cells, and consequently accumulate on electron carriers with increasingly negative redox midpoint potential. Oxygen depletion induces a transient increase in excitation towards PSI in the heterokont algae. A similar response to oxygen depletion is also present for the Viridiplantae model organism *C*. *reinhardtii*. Shifting excitation towards PSI in oxygen-free conditions is consistent with the need to oxidize a reduced photosynthetic electron transport chain. Prolonged oxygen depletion results in a clear increase in excitation directed towards PSI in *N*. *oceanica* (Fig 3B”) and a more subtle redistribution of light energy to PSII in *P*. *tricornutum* and *C*. *reinhardtii* (Fig 3A” and 3C”).

## Discussion

While *C*. *reinhardtii* is considered a model for plants concerning excitation distribution between the two photosystems, it is noteworthy that, especially in the presence of acetate in the growth medium, chlororespiration is uniquely prominent in this alga [14], a feature shared with the diatom *P*. *tricornutum* [15,16].

Exposure to high light in heterokont algae induces a redistribution of excitation energy between PSI and PSII that is opposite to the distribution pattern observed in *C*. *reinhardtii*. This regulation of excitation distribution is consistent with a high-light induced oxidation of the electron transport chain in heterokont algae. The incomplete reduction of the plastoquinone pool upon short light-pulses in heterokont algae, as employed by the saturating pulse method [21] would lead to a systematic under-assessment of the maximum fluorescence yield, and thus influence the assessment of photosynthetic performance when this technique is utilized.

### Carotenoid dependent quenching versus structural rearrangement

Are the observed changes in distribution of excitation energy between PSI and PSII in heterokont algae due to a carotenoid-specific quenching that affects light-harvesting complexes associated with PSI and PSII differently, or do light-harvesting complexes change their association between PSI and PSII? The complex dynamics of excitation distribution in high light conditions likely involve contributions by the well-characterized, pH-dependent, carotenoid-mediated excitation quenching mechanisms [10,11]. However, oxygen-depletion experiments do not change the thylakoidal pH, and application of DCMU has been shown to prevent the generation of a pH-gradient, and thus will not trigger the conversion of excitation-quenching carotenoids in *Nannochloropsis* [50] and *P*. *tricornutum* [51]. However, it has also been demonstrated that in dark-adapted green algae of *C*. *reinhardtii* [14], the prasinophycean alga *Mantoniella squamata* [52], the heterokont alga *P*. *tricornutum* [16] and *T*. *pseudonana* [53] chlororespiration can generate an oxygen-dependent pH gradient and thus potentially engage carotenoid conversion and the associated non-photochemical quenching. In addition, there is evidence that chlororespiratory activity in green algae is involved in the synthesis of carotenoids [54]. Oxygen-depletion may therefore collapse the pH-gradient, as chlororespiration is oxygen dependent, and result in the conversion to the non-quenching carotenoid and consequently cause a loss of non-photochemical quenching and an increase in chlorophyll fluorescence.

Studying the effect of high light exposure, Lepetit and co-workers [32] report the *de novo* synthesis of excitation-quenching carotenoids, which accumulate with a half time of 3–4 hours. *De novo* synthesis thus appears too slow to account for the rapid high light-induced dynamics we observe.

We observed complex spectral changes of 77 K fluorescence emission both in dark oxygen-depleted conditions and in DCMU-treated samples under high light. While abolishing chlororespiration by oxygen depletion in the dark may abolish the pH-gradient, and thus relax carotenoid-mediated non-photochemical quenching, this is not known to change energy distribution between the two photosystems. As we also observe changes in 77K fluorescence spectra in DCMU-treated cells exposed to high light, this redistribution of excitation energy appears to be independent of the thylakoidal pH and the associated carotenoid-dependent excitation quenching and may indicate a redistribution of light-harvesting systems.

### Regulation of excitation energy

In *Nannochloropsis* in anaerobic conditions, an immediate response shifts excitation energy towards PSI within 10 minutes, which is followed by a shift of excitation energy back to PSII, which is completed after about 30 minutes. These two phases likely indicate a fine-tuning of excitation distribution that is mediated by a single mechanism, or the consecutive action of two independent mechanisms. Whether these mechanisms are identical to the multistep model for non-photochemical excitation quenching in *Nannochloropsis gaditana* suggested by Chukhutsina and co-workers [55] and in *P*. *tricornutum* by Giovagnetti and Ruban [45] requires further study. Compared to Viridiplantae, the fluorescence signal indicative of this re-distribution is smaller in heterokont algae. It has recently been demonstrated that PSI-LHC complexes of heterokont algae possess trapping times that are three times shorter than those of plants [56]. Therefore, re-directing excitation from PSII to PSI will lower the overall PSII fluorescence emission, while only a small increase in PSI fluorescence is observed. This is unlike the situation in Viridiplantae, where redistribution results in higher PSI fluorescence emissions in addition to lower PSII emission, and these changes in emission provide a clear beacon for a changed distribution of excitation energy. The small changes in the magnitude of fluorescence emission in heterokont algae may have contributed to the original assessment by Owens [35] that excitation energy is not redistributed in the heterokont alga *P*. *tricornutum* in dependence of the reduction state of the plastoquinone pool. This conclusion was obtained with the assumption that the photosynthetic machinery of heterokont algae responds to light in the same manner as Viridiplantae, an assumption we can now amend.

What is the sensor that triggers the redistribution of light energy between the two photosystems? The excitation distribution induced in dark and oxygen-depleted conditions suggests that the reduction state of an electron carrier is used to modulate the distribution of excitation in heterokont algae. The plastoquinone pool is a possible candidate for this role, as it is a modulator of fluorescence, and also acts as a sensor for shifting excitation energy in cyanobacteria [57] and Viridiplantae [58].

The redox-dependent response may share components and organization with the redox-dependent pigment synthesis and synthesis of light-harvesting systems described for *P*. *tricornutum* [32]. A second sensing system may be responsible for the shift in excitation distribution induced by prolonged anaerobic conditions, where more electron carries with a more negative redox midpoint potential will become reduced. Here ferredoxins and thioredoxins are potential candidates, as these electron carriers are know to also have a redox-dependent regulatory function in carbon fixation in Viridiplantae and to a lesser extend in heterokont algae [59,60]. The slow change in excitation energy distribution during prolonged anaerobic condition could also indicate the conversion of carotenoids. Here the production availability of NADPH, may enable the conversion of carotenoids, as this electron carrier has been demonstrated to be required as a cofactor for the diatoxanthin epoxidase that is not available in dark aerobic condition in *P*. *tricornutum* [61].

### Avoiding a reduced plastoquinone pool

The photosynthetic machineries of the heterokont algae *Nannochloropsis* and *P*. *tricornutum* are tuned to avoid reduction of the photosynthetic electron transport chain during high light illumination. As this is in contrast to Viridiplantae, it is tempting to speculate on the physiological reasons for this adaptation. We propose that differences in the characteristics of the electron transport chain are based on the different light environments that terrestrial and marine organisms experience. *C*. *reinhardtii* was isolated from soil, and is thus, like Viridiplantae in general, adapted to an environment that largely lacks rapid variation in light exposure. In contrast, marine heterokont algae can experience wave lensing-induced high-light [7,8], and have therefore evolved a photosynthetic electron transport chain that is tuned to avoid high light-induced over-reduction. If environmental conditions are encountered that temporarily overwhelm the photosynthetic machinery, a build up of protons is the likely consequence, which triggers the well-characterized carotenoid-dependent energy dissipation mechanism [12]. These proposed functions of electron transport poise and xanthophyll cycle in photoprotection is supported by the ability of four *P*. *tricornutum* species to thrive in fluctuating light without increasing the amount of energy-quenching carotenoids [45,62]. In conclusion, our study provides key insights for re-interpreting previous studies and for directing future studies regarding the photosynthetic machinery and physiology of heterokont algae and their adaption to the marine environment.

## Materials and methods

### Cell cultures

*Nannochloropsis oceanica* CCAP 211/46 was obtained from the Culture Collection of Algae and Protozoa (Argyll, UK), *Chlamydomonas reinhardtii* CC-4532 MT- from the *Chlamydomonas* Resource Center at the University of Minnesota, and *Phaeodactylum tricornutum* CCMP 632 was obtained from the National Center for Marine Algae and Microbiota (East Boothbay, ME, USA).

*N*. *oceanica* and *P*. *tricornutum* were grown on f/2 medium [63] and *C*. *reinhardtii* was grown on TrisAcPO_4_ (TAP) medium [64]. Pre-cultures of the algae were incubated at 18°C and with a light intensity of 200 μmol photons m^-2^s^-1^. Before experiments, the cell cultures were harvested and either diluted with fresh medium or up-concentrated by centrifugation to a chlorophyll concentration of 1 μg Chl/mL in case of the 77 K spectroscopy experiments, and 20 μg Chl/mL (in case of *C*. *reinhardtii* and *N*. *oceanica*), and 40 μg Chl/mL (in case of *P*. *tricornutum*) for the PAM experiments. The chlorophyll concentration used for PAM experiments was adjusted to result in an oxygen consumption rate of approximately 15 μmol mL^-1^min^-1^ for each organism. Additional PAM experiments were performed for *Phaeodactylum* with a lower chlorophyll concentration of 0.2 μmol Chl/mL in order to be sure to avoid self-shading. In these additional experiments, DBMIB with a working concentration of 1 μM [32] was used to ensure a reduce plastoquinone pool. After being diluted or up-concentrated to the correct chlorophyll concentration, the cultures were dark-acclimated for one hour prior to experiments.

### PAM experiments

The response of the photosynthetic machinery to anaerobic incubation was investigated using a PAM fluorometer (Multi-color PAM, Walz, Germany) coupled to an oxygen-measuring device (Oxygraph, Hansatech, United Kingdom). This setup allowed us to record variable chlorophyll fluorescence and oxygen concentration simultaneously. A weak measuring light of 440 nm was used to assess variable fluorescence throughout the experiment, and bright light pulses of 440 nm having a duration of 0.8 s, and a light intensity of 1600 μmol photons m^-2^s^-1^ of blue light, corresponding to an effective light intensity of 3200 μmol photons m^-2^s^-1^ of white light were applied every 4 minutes. For every measurement, 2 ml of standardized cell cultures were used. In order to analyse the fluorescence decay of the recorded induction curves, a custom written MATLAB script was used [20].

### 77K experiments

For the 77 K experiments, algae were exposed to three experimental conditions: (1) high light, (2) high light in the presence of the photosystem II (PSII) inhibitor 3-(3,4-dichlorophenyl)-1,1-dimethylurea (DCMU), and (3) anaerobic conditions in darkness. Samples were dark-adapted for 1 hour in aerobic conditions, before being exposed to the three experimental conditions. In the high light + DCMU experiment, DCMU dissolved in water, having a concentration of 10 mM was added 1:1000 to algae samples, to a working concentration of 10 μM. The light intensity for the high light exposure (in presence and absence of DCMU) was 1000 μmol photons m^-2^s^-1^ of white light. In the anaerobic experiment, oxygen depletion was achieved by bubbling the algae with N_2_ gas containing 0.5% CO_2_. Aliquots of the algae were taken every 5 minutes in triplicate. In the high light experiments, one zero sample was taken first. Then, another zero sample was taken after the addition of DCMU. In the high light experiment performed without the addition of DCMU, two zero samples were taken at the same time points as the zero samples without and with inhibitor were taken in the DCMU experiment. This was done to standardize the sampling procedure. The experiment was continued for 30 minutes. The samples were frozen in liquid nitrogen immediately and stored in liquid-nitrogen-filled dewars before fluorescence measurements at 77K.

Fluorescence spectra at 77 K were acquired using a Jaz spectrophotometer (Ocean Optics, USA) in a custom designed setup [65]. The software used for recording spectra was the SpectraSuite software (Ocean Optics, USA). A 435 nm LED served as the excitation light source, and a long-pass filter was used to filter out the LED.

### Processing of 77 K data

The fluorescence spectra recorded at 77 K were processed using custom-written MATLAB scripts. First, the 77 K fluorescence spectra, which were recorded in triplicate for each experimental condition, were base line-subtracted and normalized to the wavelength corresponding to photosystem I (PSI) for each organism (Fig 2, PSI spectra shown in Fig 4). After base line-subtraction and normalization, the triplicate spectra were combined and standard deviations were calculated.

In order to generate the plots shown in Fig 3, every wavelength in the spectrum recorded at one particular time point was divided by the same wavelength in the zero spectrum in the same experiment, which, being aerobic and dark adapted, served as the reference sample. For the high light experiment that contained DCMU, dark-adapted samples 5 minutes after exposure to the herbicide were used to obtain the reference spectra. From these ratio spectra, the wavelengths found to correspond to the fluorescing components present in the respective organisms were plotted (Fluorescence ratios over time for all fluorescent components are shown in Fig 3, while the fluorescence spectra for the individual components are shown in Fig 4).

The spectra associated with different photosynthetic components were identified by normalizing all spectra in one time series to a wavelength connected to one particular component, and subsequently subtracting one spectrum in the time series from another. Using this method repetitively allowed us to eliminate the fluorescence contribution of one fluorescing component at a time. To demonstrate the approach and its validity, a step-wise example of this process is presented in S2 Fig for the *C*. *reinhardtii* experiment performed with high light. The same procedure was performed on all 77 K datasets and resulted in resolving three spectral components for *C*. *reinhardtii* and *Nannochloropsis*, and four spectral components for *P*. *tricornutum* (Fig 4). The extracted spectral components were identical for all conditions for each organism, except for a slight shift in wavelengths in the case of DCMU treatment in all organisms.

## Supporting information

S1 Fig PAM fluorometry results for *P. tricornutum* cells and previously published results for *N. oceanica* and *C. reinhardtii* for comparison. (**A, B, C**) Light pulse-induced maximum fluorescence yield (normalized to F_0_) for untreated and DBMIB-treated cells of *C*. *reinhardtii* (**A**), *N*. *oceanica* (**B**) and *P*. *tricornutum* (**C**) in dependence of light pulse intensity. The results for *C*. *reinhardtii* and *N*. *oceanica* have been published previously [20], and were included here for comparison with the newly obtained data for *P*. *tricornutum* cells at low (0.2 μM chlorophyll concentration). (**A’**, **B’**, **C’**) Chlorophyll fluorescence kinetic decay rates after light pulses of untreated and DBMIB-treated cells of *C*. *reinhardtii* (**A’**), *N*. *oceanica* (**B’**) and *P*. *tricornutum* (**C’**) in dependence of light pulse intensity. The previously published results for *C*. *reinhardtii* and *N*. *oceanica* [20] were included for comparison with newly obtained data for *P*. *tricornutum* at low (0.2 μM) chlorophyll concentration. (**A”**, **B”**, **C”**) Chlorophyll fluorescence kinetic decay rates after light pulses (1600 μmol photons m^-2^s^-1^ of blue light) of *C*. *reinhardtii* (**A”**), *N*. *oceanica* (**B”**) and *P*. *tricornutum* (**C”**) undergoing anaerobic transition. The fluorescence data behind the decay rates shown in panels **A”**, **B”** and **C”** is shown in Fig 1.
(TIFF)

S2 Fig Example of deduction of fluorescent components for *Chlamydomonas*. An example of the procedure used to isolate the spectra of fluorescing components. The dataset recorded for *C*. *reinhardtii* during high light treatment (**A**) was normalized to 675 nm (**B**). This is a wavelength that is assumed to be associated with light harvesting complexes in *C*. *reinhardtii*, and also a wavelength that is thought to be little impacted by changes in fluorescence from the PSII core complexes. To eliminate the fluorescence contribution of light harvesting complexes, one of the spectra in the time series was subtracted from the other. In this example, the t = 0_1 spectrum was subtracted from the other spectra, resulting in the spectra shown in (**C**). The next step was to neutralize the contribution of PSII. Therefore, all spectra in (**C**) were normalized to the local fluorescence maximum (in (**C**) seen as a minimum) around 687 nm, resulting in the spectra displayed in (**D**). The t = 0_2 spectrum was omitted in this panel because of it non-existent local peak at 687 nm. To compensate for the contribution of PSII to the spectra, one spectrum was chosen to be subtracted from the other spectra. This time, the t = 15 m spectrum was used, resulting in the spectra in (**E**), now thought to only to contain spectral information of PSI. The PSI fluorescence component can be obtained directly from (**E**), while the PSII fluorescence component can be calculated by subtracting the PSI component from a spectrum where the LHC component has already been subtracted (**C**, **D**). When both the PSI and the PSII fluorescence spectra are known, the fluorescence spectrum of LHCs can be found by subtracting a linear combination of the PSII and the PSI spectra from one of the raw spectra (**A**, **B**).
(TIFF)

S1 Discussion of S1 Fig Discussion of the PAM fluorometry results shown in S1 Fig. (DOCX)

S1 Dataset Raw data from PAM fluorescence experiments and 77 K spectroscopy experiments. (ZIP)
