# Supplementary figures and images for: Unique photosynthetic electron transport tuning and excitation distribution in heterokont algae
Source: PLoS One. 2019 Jan 9;14(1):e0209920. doi: 10.1371/journal.pone.0209920 (PMC6326504; doi:10.1371/journal.pone.0209920)

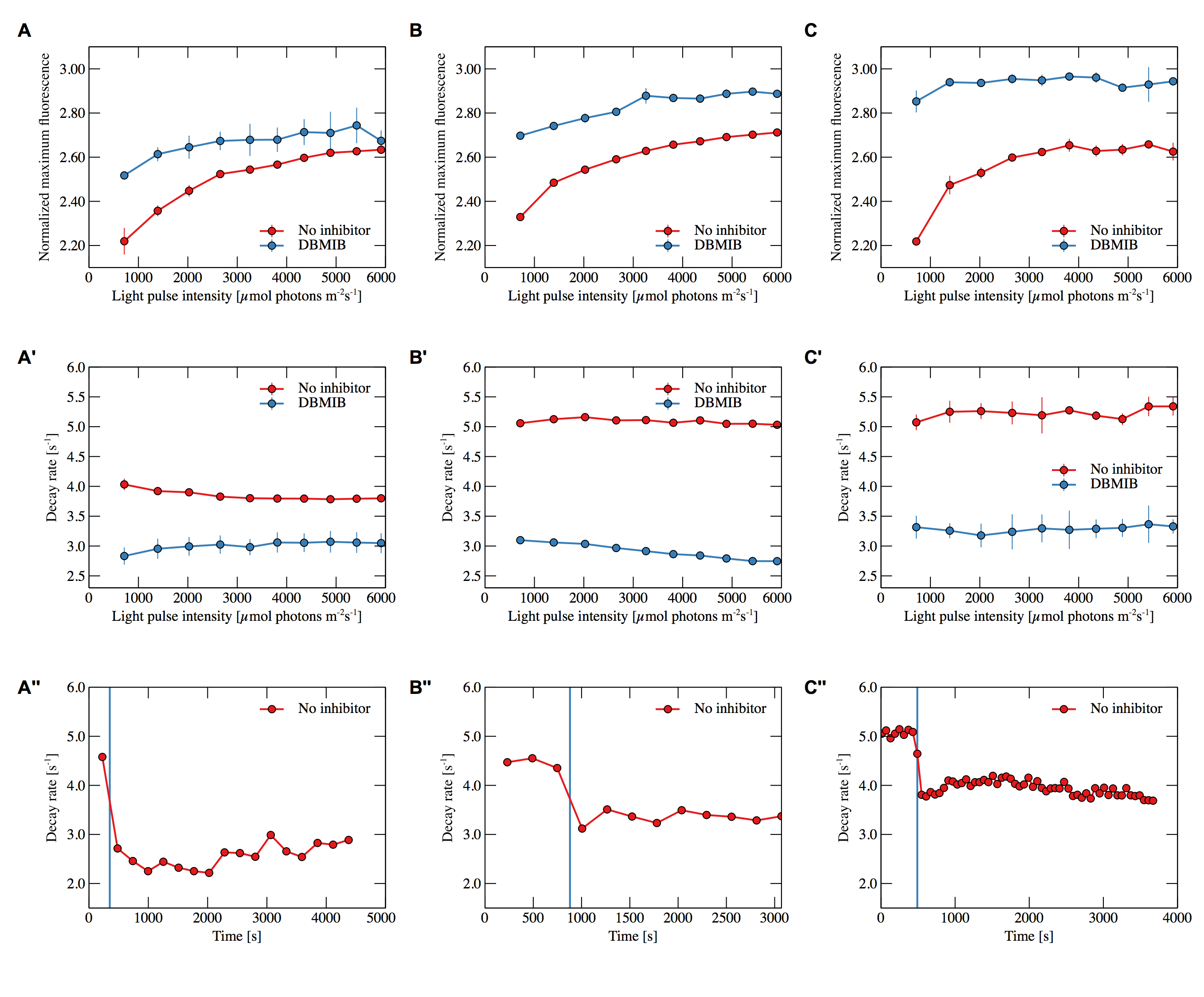

Supplement: S1 Fig — (A, B, C) Light pulse-induced maximum fluorescence yield (normalized to F0) for untreated and DBMIB-treated cells of C. reinhardtii (A), N. oceanica (B) and P. tricornutum (C) in dependence of light pulse intensity. The results for C. reinhardtii and N. oceanica have been published previously [20], and were included here for comparison with the newly obtained data for P. tricornutum cells at low (0.2 μM chlorophyll concentration). (A’, B’, C’) Chlorophyll fluorescence kinetic decay rates after light pulses of untreated and DBMIB-treated cells of C. reinhardtii (A’), N. oceanica (B’) and P. tricornutum (C’) in dependence of light pulse intensity. The previously published results for C. reinhardtii and N. oceanica [20] were included for comparison with newly obtained data for P. tricornutum at low (0.2 μM) chlorophyll concentration. (A”, B”, C”) Chlorophyll fluorescence kinetic decay rates after light pulses (1600 μmol photons m-2s-1 of blue light) of C. reinhardtii (A”), N. oceanica (B”) and P. tricornutum (C”) undergoing anaerobic transition. The fluorescence data behind the decay rates shown in panels A”, B” and C” is shown in Fig 1. (TIFF) [file pone.0209920.s001.tiff]

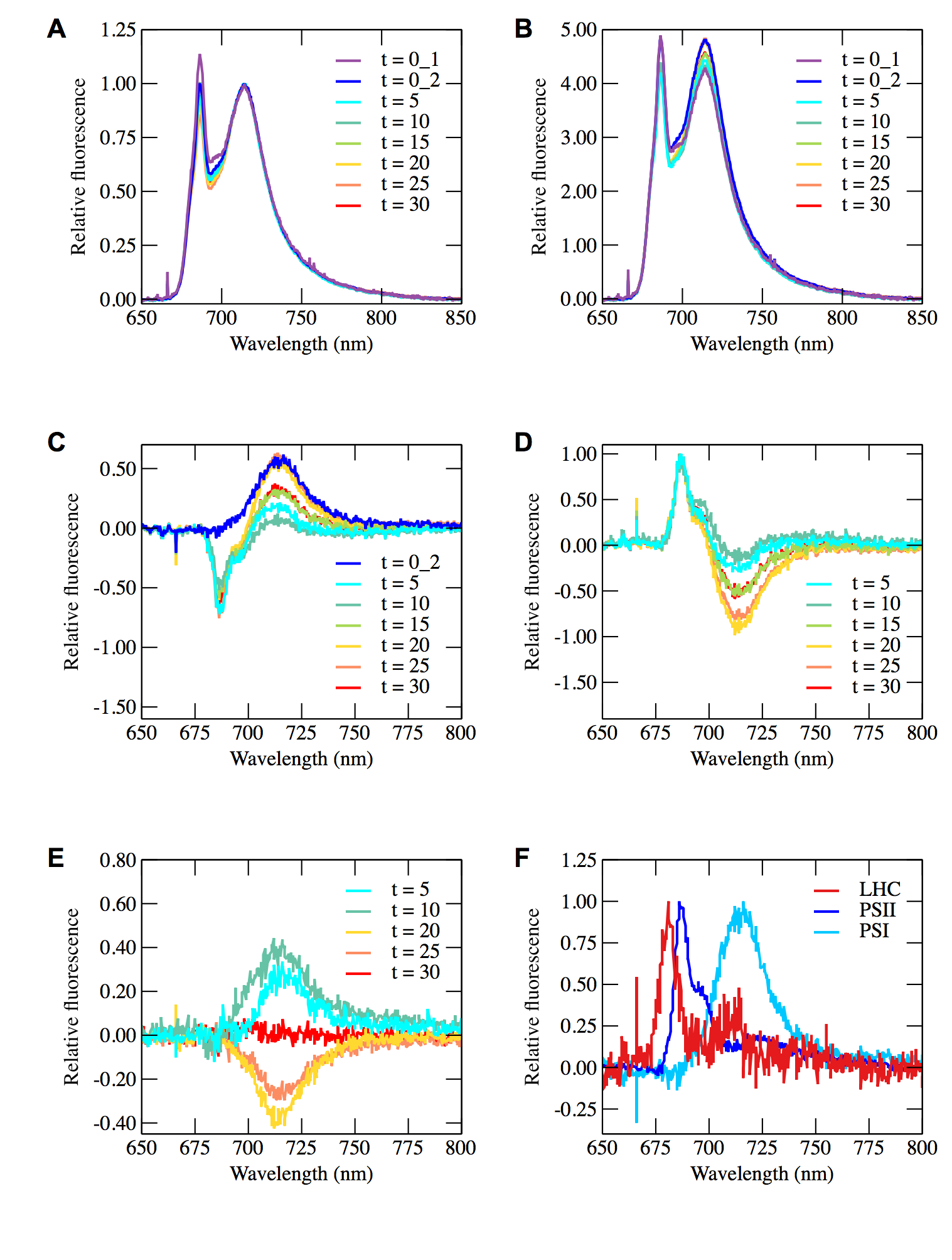

Supplement: S2 Fig — An example of the procedure used to isolate the spectra of fluorescing components. The dataset recorded for C. reinhardtii during high light treatment (A) was normalized to 675 nm (B). This is a wavelength that is assumed to be associated with light harvesting complexes in C. reinhardtii, and also a wavelength that is thought to be little impacted by changes in fluorescence from the PSII core complexes. To eliminate the fluorescence contribution of light harvesting complexes, one of the spectra in the time series was subtracted from the other. In this example, the t = 0_1 spectrum was subtracted from the other spectra, resulting in the spectra shown in (C). The next step was to neutralize the contribution of PSII. Therefore, all spectra in (C) were normalized to the local fluorescence maximum (in (C) seen as a minimum) around 687 nm, resulting in the spectra displayed in (D). The t = 0_2 spectrum was omitted in this panel because of it non-existent local peak at 687 nm. To compensate for the contribution of PSII to the spectra, one spectrum was chosen to be subtracted from the other spectra. This time, the t = 15 m spectrum was used, resulting in the spectra in (E), now thought to only to contain spectral information of PSI. The PSI fluorescence component can be obtained directly from (E), while the PSII fluorescence component can be calculated by subtracting the PSI component from a spectrum where the LHC component has already been subtracted (C, D). When both the PSI and the PSII fluorescence spectra are known, the fluorescence spectrum of LHCs can be found by subtracting a linear combination of the PSII and the PSI spectra from one of the raw spectra (A, B). (TIFF) [file pone.0209920.s002.tiff]
