# Supplementary material for: Unique photosynthetic electron transport tuning and excitation distribution in heterokont algae
Source: PLoS One. 2019 Jan 9;14(1):e0209920. doi: 10.1371/journal.pone.0209920 (PMC6326504; doi:10.1371/journal.pone.0209920)
Supplement: S1 Discussion of S1 Fig — (DOCX) [file pone.0209920.s003.docx]

**S1 Discussion of S1 Fig**

The decay kinetics of variable chlorophyll fluorescence after light pulses indicate recombination of an electron at Q_A_ with the donor side of PSII. This recombination has been shown to be dependent on the reduction state of the plastoquinone pool [66] and independent of oxygen concentration [67]. In aerobic conditions (S1 Fig 1A’’, B’’, and C’’), the plastoquinone pool is oxidized, and a high recombination rate is observed. After transition to anaerobic conditions the plastoquinone pool becomes reduced, and the recombination rates decrease, indicating a reduced plastoquinone pool. Following this argumentation, the plastoquinone pool is reduced during bright light pulses in *C. reinhardtii* (S1 Fig 1A’), while it remains oxidized in *N. oceanica* (S1 Fig 1B’) and *P. tricornutum* (S1 Fig 1C’) during bright light pulses, even at higher light intensities.

The saturating behaviour of the normalized maximum fluorescence yield (F_m_) under different light intensities indicates that the plastoquinone pool is reduced in *C. reinhardtii* at relatively low light intensities (convergence of the normalized maximum fluorescence yields of untreated and DBMIB-treated samples, S1 Fig 1A), while no clear saturation is achieved in *N. oceanica* (S1 Fig 1B) and *P. tricornutum* (S1 Fig 1C). The F_m_ for all organisms is higher in DBMIB-treated cell than in untreated cells. This is due to the differential quenching effect of DBMIB on minimum fluorescence yield (F_0_) and F_m_ [34], where F_0_ is preferentially quenched. Thus, after normalization to F_0_, the maximum fluorescence yield is higher in DBMIB-treated samples. In the presented experiments, DBMIB concentrations were used that are known to inhibit electron transport through the cytochrome *b*_6_*f* complex in different organisms. The DBMIB concentration for *C. reinhardtii* and *N. oceanica* was 20 µM, while the DBMIB concentration used for *P. tricornutum* was 1 µM. These differences in concentration are useful for demonstrating that the quenching behaviour of DBMIB does not influence the F_m_ convergence of DBMIB-treated cells and untreated cells. If this were the case, it would be expected that F**_m_** between DBMIB-treated and untreated samples would be most similar in *P. tricornutum*, where we see the largest difference in the data.

Together, fluorescence decay rates after light pulses, and F_m_ values at different light intensities indicate that the plastoquinone pool of *C. reinhardtii* is reduced at a light intensity of approximately 3000 µmol photons m^-2^s^-1^, while the plastoquinone pool remains oxidized in *N. oceanica* and *P. tricornutum* even at higher light intensities.

1. Diner BA. Dependence on the deactivation reactions of photosystem II on the redox state of the plastoquinone pool A varied under anaerobic conditions; Equilibria on the acceptor side of photosystem II. Biochim Biophys Acta. 1977;460: 247-258.
2. Laisk A, Eichelmann H, Oja V. Oxidation of plastohydroquinone by photosystem II and by dihydrogen in leaves. Biochim Biophys Acta. 2015;1847: 565-575.
